# Persistence During Childhood Problem-Solving as a Predictor of Active Suicidal Ideation During Adolescence

**DOI:** 10.1007/s10802-020-00726-4

**Published:** 2021-01-09

**Authors:** Katherine Sarkisian, Carol Van Hulle, H. Hill Goldsmith

**Affiliations:** 1 Waisman Center & Department of Psychology, University of Wisconsin-Madison 1202 W Johnson St, Madison, WI 53706, US; 2 School of Medicine and Public Health, University of Wisconsin-Madison 750 Highland Ave, Madison, WI 53726, US

**Keywords:** suicide, suicidal ideation, attention, problem-solving, twins

## Abstract

Clarifying longitudinal, behavioral predictors for adolescent suicidality could enhance prediction and treatment efforts. We examined whether childhood attentional focusing, persistence, and problem-solving behavior are associated with risk for adolescent suicidal ideation. Participants were 116 twins, 40 of whom endorsed active suicidal ideation (*i.e.,* probands), probands’ cotwins, and matched controls. We showed that higher scores on a composite measure derived at mean age 7.7 years of (1) effort and work duration during two childhood problem-solving tasks (untangling yarn and attempting to solve an unsolvable puzzle), (2) mother reported attentional focusing, and (3) observer reported persistence predicted decreased risk for suicidal ideation at mean age 14.4 years. This prediction held when comparing probands with controls (B=−1.01, SE=.38, p=.01, OR=.37) and with their cotwins (B=−.86, SE=.38, p=.02, OR=.42). Our findings indicate that childhood problem-solving behavior relates meaningfully to risk for suicidal thoughts approximately 7 years later, on average. These results underscore how longitudinal behavioral risk factors could enhance prediction and treatment of adolescent suicidal ideation.

## Introduction

Each year in the United States, approximately 4,600 individuals between the ages of 10 and 24 die by suicide, and despite ongoing research and clinical efforts, the rate of adolescent suicide continues to increase (CDC 2012; Kessler, 2005). Cross-sectional studies have revealed numerous risk factors for suicidal thoughts, suicide attempts, and suicide deaths (*e.g.,* Brent et al., 1993; Gould et al., 1998). While these studies are useful, the high frequency and climbing rate of adolescent suicide underscore the necessity of exploring novel research designs to elucidate additional risk factors for suicidality. Examining developmental precursors of suicidal ideation could complement cross-sectional findings by providing insight into whether concurrent risk factors also exert influence longitudinally. An enhanced understanding of longitudinal risk factors for suicidal ideation could improve the timing, efficacy, and specificity of interventions for distressed adolescents.

Suicidal ideation is a crucial marker of risk for subsequent more severe forms of suicidality. Among adolescents who experience suicidal ideation, 33% create a suicide plan, and 34% attempt suicide (Nock et al., 2013). The majority of suicide attempts (88%) among adolescents are preceded by suicidal ideation, making it a useful indicator of risk for suicide attempts and death by suicide in addition to being distressing in and of itself (Lewinsohn, Rohde, & Seeley, 1996). Understanding different types of influences across development that place adolescents at risk for suicidal ideation could allow preventative measures to be provided earlier and more effectively.

## Problem-Solving Ability and Risk for Suicidality

One important group of risk and protective factors for suicidality is cognitive processes and tendencies. Wenzel and Beck (2008) assert that a mixture of dispositional vulnerability factors, cognitive tendencies associated with psychiatric diagnoses, and cognitive processes specifically relevant to suicidality shape suicidal behavior. One dispositional vulnerability factor hypothesized in the cognitive model of suicide is problem-solving deficits. Wenzel and Beck (2008) contend that dispositional factors (e.g., problem-solving deficits) exert their influence by increasing stress, which can in turn increase risk for psychiatric diagnoses. However, they emphasize that dispositional vulnerability factors, such as problem-solving, must be examined using a longitudinal study design in order to clarify whether they are associated with increased likelihood of subsequent suicidality.

Individuals who are experiencing different forms of suicidality can have various difficulties with the problem-solving process, which may in turn reduce their ability to cope with challenges and changes across contexts (Reinecke, DuBois, & Schultz, 2001; Pollock & Williams, 2004; Adams & Adams, 1996). Individuals across the spectrum of suicidality severity ranging from people in the community who have suicidal thoughts to those who are psychiatrically hospitalized for suicidality display more negative attitudes toward problem-solving (D’Zurilla, Chang, Nottingham, & Faccini, 1998; Dixon, Heppner, & Anderson, 1991). In a study comparing the problem-solving attitudes and abilities of college undergraduates, non-suicidal psychiatric inpatients, and suicidal psychiatric inpatients, negative problem orientation (*i.e.,* low problem-solving self-efficacy, belief that problems are unsolvable, less realistic evaluations of significance for wellbeing) was more consistently related to hopelessness, depression, and suicide risk than problem-solving skill level. However, poorer problem-solving skills were only associated with increased risk among people who were psychiatrically hospitalized for suicidal thoughts or behaviors (D’Zurilla et al., 1998). Overall, these findings underscore the importance of carefully parsing how problem-solving beliefs and deficits relate to risk for different forms of suicidality in clinical and samples.

While existing research about the associations between problem-solving deficits and suicidal thoughts and behaviors is valuable, it is limited by the predominant use of self-report measures and the cross-sectional nature of most studies. The design of these studies precludes inferring causation and provides limited insight into the etiology of concurrent risk factors. Existing findings from prospective studies indicate that poor attentional shifting, as well as the associated tendency to ruminate, prospectively increases risk for depressive episodes (Stange, Connolly, Burke, Hamilton, Hamlat, Abramson, & Alloy, 2016). Additionally, findings from a prospective study of adolescents following psychiatric hospitalization indicate that stronger problem-solving abilities do not relate significantly to risk for subsequent suicide attempts, which highlights the importance of considering whether problem-solving deficits influence risk for suicidal thoughts and behaviors differently in clinical and non-clinical samples (Goldston, Daniel, Reboussin, Reboussin, Frazier, & Harris, 2001).

## Adolescent Neuropsychological Differences Associated with Severe Suicidality

Limited existing evidence about neuropsychological differences among suicidal adolescents suggests that specific facets of executive functioning play an important role in the development and maintenance of suicidal behaviors. In a sample of 60 adolescents admitted to a psychiatric inpatient unit, participants who were hospitalized due to severe suicidality made more errors of omission and commission on the Test of Variables of Attention (TOVA) than participants who were hospitalized for reasons other than suicidality (Horesh, 2001). Because errors of omission (*i.e.,* not responding to target stimulus when it is present) reflect inattention, and errors of commission (*i.e.,* responding to a stimulus other than the target stimulus) suggest impulsivity, this finding highlights the presence of specific neuropsychological differences among suicidal adolescents; however, adolescents who experience suicidal ideation, but have not attempted suicide, may not exhibit the same deficits (Horesh, 2001).

Results from our prior study address this gap in the literature and indicate that individuals who endorse concurrent suicidal ideation (*i.e.,* probands) can reliably be differentiated from both their cotwins and matched controls, who did not endorse suicidal thoughts, based on their increased levels of inattention (Sarkisian, Van Hulle, & Goldsmith, 2019). This previous study included all members of the current sample, as well as some additional participants from a related longitudinal twin study and used the same outcome of suicidal ideation reported during adolescence (Sarkisian et al., 2019). We examined three concurrently measured predictors of suicidal ideation: brooding, inattention, and impulsivity (Sarkisian et al., 2019). Although all three predictors were associated with increased odds of reporting suicidal thoughts when probands were compared with matched controls, inattention was the only predictor that persisted during the more stringent comparison of probands with their cotwins (Sarkisian et al., 2019).

Despite the apparent link between inattention and concurrent suicidality, no study to date has tested whether specific differences in behavioral correlates of attentional capacity, such as problem-solving behavior early in life, predict later active suicidal ideation. We chose to expand our focus to include not only attention, but also the higher order process of problem-solving because these two constructs are fundamentally interrelated. Task persistence is strongly linked to attentional control and can be conceptualized as an important manifestation of internalization of task goals and suppression of a dominant impulse in order to sustain task-oriented behavior (Lunkenheimer, Panlilio, Lobo, Olson, & Hamby, 2019). Because attention can be partly defined as the capacity to focus on tasks, task persistence can even be used as a proxy for attention (Lunkenheimer et al., 2019). By combining a relatively straightforward attentional variable with interrelated facets of higher-order problem-solving processes, we sought to provide a richer picture of possible indicators of cognitive and behavioral risk and resilience to subsequent suicidality.

## Hypotheses

We sought to determine whether higher levels of effort during childhood problem-solving tasks, mother reported attentional focusing, and observer reported persistence during problem-solving predict decreased risk for active suicidal ideation during adolescence. Longitudinal examinations of suicidality that include behavioral measures, rather than entirely self or caregiver report, are rarely conducted, and this is the first study of this nature in a community sample. We hypothesized that higher levels of attentional focusing and problem-solving effort, as measured by a temperament assessment battery and mother and observer reports during childhood, would predict significantly lower likelihood of endorsing suicidal ideation during adolescence. We predicted that this finding would exist in the less stringent comparison of probands with matched controls and persist in a comparison of probands with their cotwins. The proband vs. cotwin contrast is more rigorous because it inherently controls for additional shared environmental and biological characteristics (*e.g.,* neighborhood, parents’ political and religious beliefs, prenatal environment) beyond the demographic characteristics shared by probands and matched controls.

## Method

### Participants and Procedures

The sample included 116 identical (32%) and fraternal (23% same-sex; 45% opposite-sex) twins drawn from a longitudinal, community-based sample (please see Lemery-Chalfant, Goldsmith, Schmidt, Arneson, & Van Hulle, 2006 for a full description of the full sample). The sample was 46% female. Forty of the twins in the sample endorsed suicidal ideation (*i.e.,* probands), so the sample is comprised of 40 probands, 40 matched controls, and 36 cotwins of probands. In two twin pairs, both twins endorsed suicidal thoughts, so these four twins were all classified as probands (*i.e.,* no unaffected cotwin). The full sample was recruited using birth records and mildly enriched for psychopathology during middle childhood (*i.e.,* approximately age 7; at least one member of the pair scored at least 1½ standard deviations above the mean level of depression, anxiety, overanxiousness, oppositional defiance, aggression, conduct disorder, inattention, or impulsivity reported by participants’ parents; cotwins of participants above at least one of these cutoffs and twin pairs in which neither member was above these cutoffs were included, as well; Schmidt, Van Hulle, Brooker, Meyer, Lemery-Chalfant, & Goldsmith, 2013). Probands’ matched controls were selected from the full longitudinal twin sample (N=1,140) based on the degree to which they resembled probands’ demographic characteristics, including sex (45% female for probands and controls), age (proband mean age=14.3 years; controls mean age=14.6 years), ethnicity (probands=70% Caucasian, 17.5% Black; controls=85% Caucasian, 10% Black; ethnicity was not reported for 5% of probands), family income (proband mean=$45,001-$50,000; control mean=$50,001-$60,000), and parents’ marital status (45% of probands’ biological parents married to each other vs. 65% for controls).

The probands in this subsample were significantly more diverse than the full sample in terms of ethnicity, family income, and parents’ marital status. Fifty-five percent of probands’ parents were not married to each other (*e.g.,* divorced, widowed), which differs significantly from the rest of the full sample in which 90% of participants’ parents are married to each other (Van Hulle, Lemery-Chalfant, & Goldsmith, 2007). While 86% of the sample in our full longitudinal study is Caucasian, 18% of probands are Black, 5% are Native American, and 3% are Chinese (Schmidt et al., 2013). Additionally, the mean income of probands’ families at the time of the risk assessment ($45,001 to $50,000) was somewhat lower than the mean of $50,000 to $60,000 in the full longitudinal sample. These differences highlight the necessity of inherently controlling for demographic characteristics by selecting matched controls based on them, as we did.

Informed assent was obtained from all twins included in the study, and informed consent was obtained from participants’ caregivers. The Institutional Review Board at the University of Wisconsin—Madison approved the protocol.

### Measures

#### Diagnostic Interview

Participants completed a structured diagnostic interview conducted by trained interviewers during adolescence (mean age=14.4 years). Two different diagnostic interviews were used during different phases of the study, but they assess the same core diagnostic content; both are reliable, valid, and based on DSM-IV criteria (Lucas et al., 2001). The validity, interrater reliability, and test-retest reliability of the DISC interview with children over age 9 is well-validated (*e.g.,* Piacentini, Shaffer, Fisher, Schwab-Stone, Davies, & Gioia, 1993; Schwab-Stone, Fisher, Piacentini, Shaffer, Davies, & Briggs, 1993). 31 participants completed the DISC Predictive Scales, and 75 participants completed the Diagnostic Interview Schedule for Children (DISC; Version IV; Shaffer et al., 2000). The DISC Predictive Scales (DPS) are derived from stem questions on the DISC and predict DISC diagnoses accurately (Lucas et al., 2001). The DPS contains stem items for each diagnostic domain derived from the DISC, and its shortened format is not associated with significant reductions in discriminatory power (Lucas et al., 2001). The frequencies of diagnoses for probands, cotwins, and controls are presented in Table 1. Rates at which adolescents endorsed suicidal thoughts on the DPS and DISC were quite similar (3% for DPS and 5% for DISC).

#### Suicidal Ideation

Both diagnostic interviews include an item about suicidal ideation the requires participants to answer whether they have “thought seriously about killing [her/himself]”. Phrasing for this item is identical between the two interviews aside from specific ideation time frames. Because the suicidal ideation item phrasing indicates active suicidal ideation (i.e., as opposed to passive suicidal ideation), our references to “active” ideation refer to the implied likelihood of some degree of suicidal intent, and this terminology is not meant to imply any specific time frame. The DISC asks whether this has occurred in the past year, while the DPS assesses whether it has happened in the past three months. Despite this time frame difference, rates of endorsement of suicidal ideation are similar between the two interviews (3% for DPS and 5% for DISC). Individuals were classified as probands based on whether they replied affirmatively when asked about suicidal thoughts. Individuals who were classified as cotwins or controls denied suicidal ideation.

If a participant replied affirmatively to the suicidal ideation question, or to a question about prior suicide attempts, an Institutional Review Board-approved suicide risk assessment was conducted. This assessment allowed us to determine whether participants were at imminent risk of harming themselves (*i.e.,* likely to act on thoughts of suicide within 24 hours) and whether they had a suicide plan and the means necessary for acting on it. All probands in the sample were classified as not being at imminent risk.

Notably, none of the participants in the proband, cotwin, or control groups reported any form of suicidal ideation or behavior during childhood based on caregiver reports via the DISC interview).

#### Early Measures of Problem-Solving Behavior, Persistence, and Attentional Focusing

##### Lab-TAB

Problem-solving tendencies were assessed around age 8 (mean age=7.7 years) using two episodes from Goldsmith & Rothbart (1999)’s Laboratory Temperament Assessment Battery (Lab-TAB) conducted at participants’ homes. This battery is designed to elicit a wide range of behavioral and emotional responses that is subsequently coded for relevant variables using videotapes taken at the visit. Two trained experimenters administered 14 episodes at each home visit. During the first problem-solving episode (“Tangoes”), participants were given two difficult puzzles to complete and told by the experimenter that they had all of the pieces necessary to complete each puzzle; they were unaware that the puzzles were “unsolvable” because an extra piece was included with each puzzle. During the second problem-solving episode (“Yarn Tangle”), participants were given a knotted ball of yarn and instructed to untangle it so that other children could use it for a project. Both episodes lasted for five minutes and were coded in 10-second epochs. Coded variables included the child’s overall effort, which ranged from 0 (“low effort”) to 2 (“high effort”), and duration of on-task work (*i.e.,* scored for each instance of on-task work) in each episode. Coding was completed by a team of experienced and thoroughly trained coders supervised by a master coder. Before coders could rate episodes independently, they were required to achieve at least 80% agreement with a master coder on each variable in the episode. All episodes were coded by multiple raters. Mean interrater reliabilities (i.e., Kappa) for overall effort were .84 for Tangoes and .78 for Yarn Tangle; interrater reliabilities for duration of on-task work were not calculated, as this variable is more easily observed (i.e., percent agreement between coders was approximately 89%). We combined ratings within each variable across each episode (*i.e.,* as a sum for duration of on-task work or as a mean for overall effort).

### Behavioral Observations

Following each visit, the two experimenters completed a global rating of each twin’s behavioral and affective tendencies, including persistence. The persistence scale assessed each twin’s persistence when attempting to complete tasks and ranged from 1 (“consistently lacks persistence; stops or gives up before task is completed”) to 4 (“consistently persistent; never quits”). We averaged the two experimenter ratings to create a mean observed persistence score. Interrater reliability was moderate (Kappa=.41; ICC=.79), which is relatively unsurprising given the more abstract nature of behaviorally observed persistence.

### Parent Ratings

Participants’ mothers completed the Children’s Behavior Questionnaire (CBQ) at the time of the in-home temperament assessment. The CBQ assesses multiple temperament domains during childhood, including attentional focusing (Rothbart, Ahadi, Hershey, & Fisher, 2001). The CBQ responses scale ranges from 1 (“extremely untrue”) to 7 (“extremely true”). The Attentional Focusing scale of the CBQ includes 14 items, (*e.g.,* “When drawing or coloring in a book, shows strong concentration”). We used a modified version of this scale that included 10 of the 14 items and had an internal consistency (i.e., Cronbach’s alpha) score of .76. The shortened CBQ, also used in other projects, reduced participant burden, while maintaining sufficient items for each scale to cover the breadth of the construct and retain strong psychometric quality.

### Data Analysis

After descriptive analyses including examination of gender differences, we then used logistic regression analyses to examine whether higher levels of attentional focusing, persistence, and intensity and duration of effort during childhood problem-solving (*i.e.,* individual behavioral, observer reported, and mother reported variables and a composite of behavioral and mother and experimenter reported attentional focusing and persistence) were associated with significantly decreased risk of endorsing active suicidal ideation during adolescence. The inclusion of three separate sources of information about participants’ problem-solving behavior provided us with a rich picture of participants’ tendencies across different settings. In all analyses, we used a significance cutoff of p<.05 (two-tailed). SPSS (version 22) was used for all analyses (SPSS, 2013).

Our analyses included two group comparisons. First, we compared probands to matched controls; this comparison allowed us to control for demographic variables shared by probands and their controls (*i.e.,* sex, age, family income, parents’ marital status, and ethnicity) when testing whether duration and intensity of problem-solving effort during childhood were related to subsequent suicidal ideation. Our second analysis compared probands and their cotwins. Probands and their cotwins share not only basic demographic characteristics, but also other unmeasured biological and environmental influences (*e.g.,* prenatal environment, neighborhood, parents’ political views), thus providing a more stringent test of childhood problem-solving behavior because we are implicitly controlling for more biological and environmental factors shared by twins.

Finally, we extended the logistic regression approach to examine whether the associations between early problem-solving behavior and risk for adolescent suicidal ideation were attributable to comorbid ADHD or depression.

### Missing Data

Problem-solving task data were missing for a substantial portion of probands (~35%). More than two times as many probands (and their cotwins) as matched controls were missing data from the in-home temperament assessment due to planned missingness in data collection. Minority families in particular were invited to participate in a shortened version of the age 7 assessment that lacked the home visit component. This decision was based on logistical concerns inherent with collecting data from cities further away from the relatively ethnically homogeneous city in which our laboratory is located. Because individuals belonging to ethnic minorities comprise a significant portion (approximately 20%) of the sample, particularly our proband and cotwin groups, they were missing data at higher rates. In addition, some families, including some probands, were recruited during later phases of the study. Due to these nonrandom patterns of missingness, we elected not to impute any missing values.

A few participants (9%) were missing adolescent diagnostic data. Five probands, and four of their cotwins, did not complete the diagnostic assessment due to family distress associated with suicidal ideation endorsement. Any other missing data for diagnostic variables appears unrelated to study outcomes. This missingness is below the 10% threshold that would suggest likely bias with the complete data approach (Bennett, 2001).

## Results

### Suicidal Behavior

We used responses from the risk assessment to characterize complex aspects of probands’ suicidality beyond imminent vs. non-imminent risk and presence or absence of ideation and prior attempt(s). When asked if they had a suicide plan, 13% of probands responded affirmatively. We then asked participants how likely it was that they would act on their thoughts of suicide; 20% reported essentially zero likelihood (*e.g.,* “I would never do it” or “zero percent chance”), 45% reported low to moderate likelihood (*e.g.,* “not likely”), and 18% reported higher or unclear likelihood (*e.g.,* “I’m not sure” or “5 out of 10”). Five participants were unable to clarify the likelihood that they would act on their thoughts, and answers given by two participants were either lost or not recorded initially.

### Means, Variability, and Sex Differences for the Predictor Variables

The means and variability for the problem-solving episode variables (*i.e.,* overall effort and duration of on-task work for each of the two episodes), observer reported persistence, and mother-reported attentional focusing, and are listed in Table 2.

In the combined sample of probands, cotwins, and matched controls, no significant sex differences occurred for any problem-solving, attentional focusing, or persistence measures. When we examined sex differences within each group, the only significant sex difference was in probands’ mother-reported attentional focusing, which was higher among female probands than male probands (Cohen’s d=.94).

### Intercorrelations of the Predictor Variables

Next, we examined the correlations among the problem-solving episode variables, mother-reported attentional focusing, and observer reported persistence. As expected, the two variables from each of the problem-solving episodes correlated highly (*i.e.,* .86 for Yarn Tangle overall effort and duration of on-task work, p<.001; .90 for Tangoes overall effort and duration of on-task work, p<.001; Table 3). Effort and on-task work duration variables also correlated fairly highly between episodes (*i.e.,* .52 for Yarn Tangle and Tangoes overall effort variables, p<.001; .46 for Yarn Tangle and Tangoes duration of on-task work variables, p<.001; Table 3). Additionally, both mother-reported attentional focusing and observer-reported persistence correlated significantly with all other predictor variables with the exception of the nonsignificant correlation between observer-reported persistence and mother-reported attentional focusing (r=.10, p=.40). Overall, these generally significant correlations support our decision to combine these six predictors into one problem-solving composite (Table 3). Additionally, we conducted a principal components analysis, which indicated only one component.

### Childhood Problem-Solving Behavior as a Predictor of Adolescent Suicidal Ideation (Matched Control Analysis)

We then tested the combined childhood problem-solving composite (*i.e.,* included 4 behavioral variables, 1 mother reported variable, and 1 observer reported variable) as a predictor of adolescent suicidal ideation (Table 4). This multifaceted composite accounted for 19% of the variance in adolescents’ endorsement of active suicidal ideation, and the model classified 65% of probands and controls correctly. The predicted likelihood of an adolescent reporting suicidal ideation decreased by a factor of .37 for each one standard deviation increase in this composite of mother-reported attentional focusing, observer reported persistence, and intensity and duration of problem-solving effort (B= −1.01, p=.008). In general, patterns of results involving the individual components of this composite paralleled these findings (Table 5).

### Childhood Problem-Solving Behavior as a Predictor of Adolescent Suicidal Ideation (Cotwin Analysis)

We used a binary logistic regression model to test whether the childhood problem-solving composite was associated with adolescent suicidal ideation among probands and their cotwins (Table 4). This predictor accounted for 17% of the variance in suicidal ideation endorsement, and the model classified 62% of the probands and cotwins correctly. For each one standard deviation increase in the composite of mother-reported attentional focusing, observer reported persistence, and behaviorally measured problem-solving effort intensity and duration during childhood, the probability of an adolescent endorsing active suicidal ideation decreased by a factor of .42 (B=−.86, p=.02). Again, the individual components of this composite related similarly to subsequent suicidal ideation risk (Table 5).

### Examination of the Impact of Comorbid Attention-Deficit/Hyperactivity Disorder and Depression

To determine whether associations between early problem-solving behavior and risk for adolescent suicidal ideation were attributable to comorbid ADHD or depression, we used binary logistic regression. However, our pattern of results was not altered by controlling for concurrent ADHD and comparing probands with matched controls (B_problem-solving_= −1.16, p=.006; B_ADHD_= 1.69, p=.075. The cotwin comparison model did not converge due to the low rate of ADHD among probands’ cotwins. It did converge when controlling for concurrent depression and comparing probands with matched controls (B_problem-solving_= −1.22, p=.004, B_depression_=2.35, p=.051). Again, the cotwin comparison model did not converge due to low rate of depression among probands’ cotwins. Notably, childhood problem-solving effort level was a stronger predictor of suicidal thoughts than more temporally proximal depression or ADHD in both models that converged.

To explore this point further, we re-ran our central analyses while controlling for trained observers’ reports of depression-related behaviors seen throughout the home visit completed during childhood (*i.e.,* mean of two reporters’ impressions, like our observer-reported persistence variable). Adding this variable to the analyses did not alter our central results in the comparisons of probands with controls (B_problem-solving_= −1.41, p=, p=.009, B_depression_=−1.05, p=.44) or cotwins (B_problem-solving_= −.98, p=, p=.044, B_depression_=−1.37, p=.29).

## Discussion

Given the increasing rates of death by suicide among adolescents in the United States, examining longitudinal predictors of suicidality is crucial for informing the creation and implementation of evidence-based early interventions (CDC, 2012; Kessler et al., 2005). We examined a composite including mother-reported attentional focusing, observer-reported persistence, and intensity and duration of effort during childhood problem-solving tasks as a predictor of adolescent active suicidal ideation in a combined sample of probands who endorsed suicidal ideation as adolescents, their cotwins, and matched controls. Whether probands were compared with matched controls or cotwins, higher problem-solving composite scores (*i.e.,* higher levels of effort and duration of work during problem-solving tasks, attentional focusing, and persistence) were associated with a significant decrease in likelihood of endorsing suicidal ideation during adolescence.

### Unique Benefits of the Twin Comparison Approach

Comparing probands with their cotwins (vs. matched controls) provides a more stringent test of the association between problem-solving behavior early in life and later suicidal ideation because it inherently controls for unmeasured confounders shared by cotwins. These include not only basic demographic factors shared by probands and matched controls, but also additional biological and environmental factors (*e.g.,* neighborhood, parents’ political or religious beliefs). Moreover, analyses comparing probands with their cotwins account for all shared genetic and environmental factors (*i.e.,* measured *and* unmeasured) that might account for the link between higher levels of problem-solving persistence and decreased risk for subsequent suicidal ideation. The greater confidence in causal inference permitted by these analyses suggests that problem-solving behavior may have a causal effect on risk for subsequent suicidal ideation above and beyond shared influences, including genetic liability, family stress, and dysfunctional parenting. The consistency of the association between higher levels of problem-solving persistence during childhood and lower risk for suicidal thoughts during adolescence and its relationship to prior findings are important to explore in greater detail because longitudinal risk factors, particularly those that might exert causal influences, for adolescent suicidality are generally unclear yet important to clarify.

### Longitudinal Association between Decreased Effort During Childhood Problem-Solving and Adolescent Suicidality

Although prior studies show relatively consistent associations between problem-solving deficits and severe concurrent suicidality (*e.g.,* D’Zurilla et al., 1998), no other study has investigated whether these problem-solving deficits predict risk for suicidal thoughts years later. Existing studies of the temporally proximal links between problem-solving difficulties and suicidality risk are valuable, but they do not distinguish the two potential interpretations of this association: (1) being suicidal produces alterations in neuropsychological functioning that impede problem-solving; and (2) difficulties with problem-solving exist before suicidality emerges and increase risk for subsequent suicidal thoughts and behavior.

Markedly different prevention and intervention strategies may be indicated by these two possible interpretations, so clarifying the nature of the association between decreased problem-solving effort and suicidality is crucial. Although the probands in our sample experienced suicidal thoughts during adolescence, they were not suicidal (*i.e.,* as reported by caregivers via DISC interview that included suicidality items) when their problem-solving effort level was assessed during middle childhood. Because the probands displayed decreased duration and intensity of effort during these assessments prior to the emergence of suicidality, these deficits are likely to be precursors of suicidal ideation, rather than a neuropsychological ramification of being suicidal.

Although the longitudinal association between decreased effort during problem-solving early in life and increased risk for suicidal thoughts during adolescence suggests that these alterations in behavior precede suicidality, we cannot rule out the possibility of bidirectional effects. The two possible interpretations of extant findings outlined above are not mutually exclusive, and differences in problem-solving among suicidal individuals likely represent a confluence of trait vulnerability and state related deficits. The relative influences of trait and state related risk, as well as the precise nature of problem-solving differences (e.g., negative problem orientation vs. problem-solving skill deficits) may vary across the spectrum of suicidality severity. However, our findings do contradict prior assertions that problem-solving deficits among individuals who experience suicidality are purely state dependent (e.g., Schotte, Cools, & Payvar, 1990).

### Implications & Limitations

Our findings highlight the utility of evaluating longitudinal predictors of suicidality in addition to examining more proximal risk factors. The association between problem-solving behavior and suicidal ideation risk across a time period of approximately seven years underscores that risk factors early in life can relate in meaningful ways to subsequent suicidality. Moreover, the consistent link between our non-diagnostic, *behavioral* measures of problem-solving and risk for later suicidal ideation removes the association from the confounds inherent in parental and self-report.

Evidence of a longitudinal association between problem-solving effort level and subsequent likelihood of reporting suicidal thoughts could be translated into early intervention studies following extension and replication. Clearly, only a minority of children who display lower persistence, attentional focusing, and effort during problem-solving go on to experience suicidality, so clarifying which individuals are at greatest risk is an important goal. Further studies of more precise mechanisms through which problem-solving behavior relates to suicidal ideation risk will presumably provide more specific targets for intervention, such as negative problem orientation or deficits in at least one of the steps of rational problem-solving skills targeted by problem-solving therapy (Bell & D’Zurilla, 2009).

Elucidation of specific aspects of problem-solving that can increase risk for suicidal thoughts could make it possible to differentiate children who display low problem-solving effort but are not at risk for suicidality from those whose problem-solving attitudes or skill deficits place them at risk. At least three intervention modalities, including cognitive behavioral therapy, dialectical behavior therapy, and mindfulness-based interventions, could be used to help children learn to identify and modify problematic attitudes toward problem-solving and ineffective approaches. Problem-solving therapy, a form a cognitive behavioral therapy, may be a particularly promising option, as it is designed to specifically target problem-solving attitudes and abilities (D’Zurilla & Nezu, 1999).

Our study has several limitations. We were unable to determine whether childhood problem-solving behavior predicts increased risk for suicide attempts or death by suicide due to a low number of suicide attempts and no known deaths by suicide. This analytic plan was determined a priori due to the relatively low number of suicide attempts in our sample. The longitudinal nature of our data permits some additional inferences regarding the potentially causal effects of early problem-solving tendencies on subsequent suicidal ideation risk compared with cross-sectional analyses, but the potential effects of confounding variables still preclude firm inferences about causality. Additionally, more frequent assessments of the course of problem-solving skill development and emergence of suicidality would likely have offered further insight into these developmental processes. Another limitation of our study is that we did not explicitly assess problem solving orientation or problem-solving ability level, so it is difficult to discern whether one or both of these components drive the association between decreased problem-solving effort and adolescent suicidal ideation. More generally, we note that although the process of problem-solving can be conceptualized as having four main components (*i.e.,* problem definition and formulation, generation of alternatives, decision making, and solution implementation and verification; Bell & D’Zurilla, 2009). Our measures did not assess these components individually, primarily because the measures were part of a comprehensive temperament assessment battery that was designed to survey a wide range of tendencies and traits. We combined behavioral data with mother and observer reports to provide the richest picture of problem-solving permitted by our dataset, but we acknowledge portrayal of problem-solving is primarily in terms of focusing and persistence. In addition, although our sample is representative of the population of Wisconsin, it is not necessarily representative of other regions, which may impact generalizability. Relatedly, although we matched proband with controls as well as we could and prioritized demographic variables that seemed most important to match on (i.e., sex, age), this resulted in some less than ideal matches on other demographic variables like ethnicity, family income, and parents’ marital status due to the relatively demographically homogeneous nature our sample. However, our use of cotwin controls overcomes this limitation.

Although our study has some limitations, our results help to clarify one way in which suicidal ideation during adolescence can be predicted using childhood behavior, which is a generally unexplored domain of suicide research. Studies of suicidality rarely examine factors involved in the emergence of less severe suicidal behavior, making it difficult to design and deliver early interventions for at-risk individuals in an evidence-based manner. Exploring associations between early markers of vulnerability and suicidal thoughts later in life is essential because it can provide concrete targets for interventions designed to help distressed children and adolescents who are at risk for suicidal thoughts and behavior sooner and more effectively.

## Figures and Tables

**Table 1.** Frequency of diagnoses by group.

	Probands (%)	Cotwins of Probands (%)	Matched Controls (%)
Major depressive disorder	8 (20%)	0	0
Social anxiety disorder	4 (10%)	1 (2.8%)	3 (7.5%)
Separation anxiety disorder	7 (17.5%)	2 (5.6%)	1 (2.5%)
Agoraphobia	5 (12.5%)	4 (11.1%)	2 (5%)
Panic disorder	5 (12.5%)	5 (13.9%)	2 (5%)
Generalized anxiety disorder	6 (15%)	1 (2.8%)	1 (2.5%)
Specific phobia	11 (27.5%)	9 (25.6%)	7 (17.5%)
Obsessive-compulsive disorder	9 (22.5%)	3 (8.4%)	2 (5%)
Post-traumatic stress disorder	10 (25%)	5 (13.9%)	2 (5%)
Eating disorder (anorexia nervosa or bulimia nervosa)	9 (22.5%)	5 (13.9%)	1 (2.5%)
Attention deficit-hyperactivity disorder	8 (20%)	2 (5.6%)	3 (7.5%)
Oppositional defiant disorder	4 (10%)	3 (8.3%)	5 (12.5%)

*Note.* Diagnoses are based on Diagnostic and Statistical Manual of Mental Disorders, 4^th^ Edition (DSM-IV) criteria. Proportions of each group with each diagnosis are based on the number of individuals with complete diagnostic data *not* the total number of individuals in each group.

**Table 2.** Means and standard deviations for predictor variables.

	Probands			Cotwins of Probands			Matched Controls		
	N	Mean	Standard Deviation	N	Mean	Standard Deviation	N	Mean	Standard Deviation
Yarn Tangle Overall Effort	21	−.43	1.16	19	.15	.93	31	.20	.86
Yarn Tangle Duration On-Task Work	20	−.28	1.15	19	.11	1.01	31	.12	.88
Tangoes Overall Effort	22	−.46	1.41	20	.13	.60	31	.25	.75
Tangoes Duration On-Task Work	22	−.61	1.55	20	.14	.51	31	.34	.40
Mother-Reported Attentional Focusing	27	−.47	.97	23	.37	1.05	34	.12	.87
Observer Reported Persistence	22	−.51	1.01	20	.08	1.01	31	.31	.86
Problem-solving composite	29	−.47	1.03	25	.22	.92	34	.19	.63

*Note.* All non-composite variables in the table were standardized within the total sample (i.e., z-scored). Ns reflect the number of individuals from each group with complete data.

**Table 3.** Correlations between predictor variables

	Yarn tangle mean overall effort	Yarn tangle duration of on-task work sum	Tangoes mean overall effort	Tangoes duration of on-task work sum	Persistence (home visit observer report mean)	Attentional focusing (mother report)
Yarn tangle mean overall effort	1 (n=71)					
Yarn tangle duration of on-task work sum	.86* p<.001	1 (n=70)				
Tangoes mean overall effort	.52* p<.001	.41* p<.001	1 (n=73)			
Tangoes duration of on-task work sum	.59* p<.001	.46* p<.001	.90* p<.001	1 (n=73)		
Persistence (home visit observer report mean)	.63* p<.001	.57* p<.001	.49* p<.001	.52* p<.001	1 (n=73)	
Attentional focusing (mother report)	.39* p=.001	.40* p=.001	.26* p=.03	.38* p=.001	.10 p=.40	1 (n=84)

*Note.* All composites were standardized within the full sample. In the “Yarn Tangle” task, participants were asked to untangle a ball of yarn. In the “Tangoes” task, participants were asked to work on puzzles that, unbeknownst to them, were unsolvable;

* =p<0.05.

**Table 4.** Results from logistic regression analyses with problem-solving composite as a predictor of suicidal ideation.

	Matched Control Comparison Analysis				Cotwin Comparison Analysis			
	B	Standard error	p-value	Likelihood of endorsing suicidal ideation decreased by a factor of:	B	Standard error	p-value	Likelihood of endorsing suicidal ideation decreased by a factor of:
Problem-solving behavior composite	−1.01	.38	p=.01	.37	−.86	.38	p=.02	.42

*Note.* N ranged from 25–34 in each group; see Table 2 for details. Odds ratio values are unadjusted.

**Table 5.** Results from logistic regression analyses with problem-solving effort and duration, mother-reported attentional focusing, and observer reported persistence as predictors of suicidal ideation.

	Matched Control Comparison Analysis				Cotwin Comparison Analysis			
	B	Standard error	p-value	Likelihood of endorsing suicidal ideation decreased by a factor of:	B	Standard error	p-value	Likelihood of endorsing suicidal ideation decreased by a factor of:
Yarn tangle mean overall effort	−.63*	.31	p=.040	.54	−.66	.36	p=.066	.52
Yarn tangle duration of on-task work sum	−.41	.30	p=.174	.67	−.43	.34	p=.209	.65
Tangoes mean overall effort	−.62*	.29	p=.034	.54	−.61	.34	p=.071	.54
Tangoes duration of on-task work sum	−1.07*	.46	p=.021	.34	−.72	.37	p=.052	.49
Persistence (home visit observer report mean)	−.97*	.36	p=.007	.38	−.55	.34	p=.105	.58
Attentional focusing (mother report)	−.71*	.31	p=.021	.49	−1.06*	.44	p=.015	.35

*Note.* All variables are standardized. Variables were standardized in the full sample, which is why the standard deviations are not equal to 1. In the “Yarn Tangle” task, participants were asked to untangle a ball of yarn. In the “Tangoes” task, participants were asked to work on puzzles that, unbeknownst to them, were unsolvable;

* =p<0.05. N ranged from 22–34 for each group; see Table 2 for details. Odds ratio values are unadjusted.
